# *In-Situ* Optical and Acoustical Measurements of the Buoyant Cyanobacterium *P. Rubescens*: Spatial and Temporal Distribution Patterns

**DOI:** 10.1371/journal.pone.0080913

**Published:** 2013-11-26

**Authors:** Hilmar Hofmann, Frank Peeters

**Affiliations:** Environmental Physics, Limnological Institute, University of Konstanz, Konstanz, Germany; Belgian Nuclear Research Centre SCK/CEN, Belgium

## Abstract

Optical (fluorescence) and acoustic *in-situ* techniques were tested in their ability to measure the spatial and temporal distribution of plankton in freshwater ecosystems with special emphasis on the harmful and buoyant cyanobacterium *P. rubescens*. Fluorescence was measured with the multi-spectral FluoroProbe (Moldaenke FluoroProbe, MFP) and a Seapoint Chlorophyll Fluorometer (SCF). *In-situ* measurements of the acoustic backscatter strength (ABS) were conducted with three different acoustic devices covering multiple acoustic frequencies (614 kHz ADCP, 2 MHz ADP, and 6 MHz ADV). The MFP provides a fast and reliable technique to measure fluorescence at different wavelengths *in situ*, which allows discriminating between *P. rubescens* and other phytoplankton species. All three acoustic devices are sensitive to *P. rubescens* even if other scatterers, e.g., zooplankton or suspended sediment, are present in the water column, because *P. rubescens* containing gas vesicles has a strong density difference and hence acoustic contrast to the ambient water and other scatterers. After calibration, the combination of optical and acoustical measurements not only allows qualitative and quantitative observation of *P. rubescens*, but also distinction between *P. rubescens*, other phytoplankton, and zooplankton. As the measuring devices can sample *in situ* at high rates they enable assessment of plankton distributions at high temporal (minutes) and spatial (decimeters) resolution or covering large temporal (seasonal) and spatial (basin scale) scales.

## Introduction

Cyanobacteria are important constituents of phytoplankton communities and ubiquitous in lakes of different nutritional status [1]. In recent years, the filamentous cyanobacterium *Planktothrix rubescens* (*P. rubescens*) has become abundant in many pre-alpine lakes [2], [3], [4]. Depending on its abundance *P. rubescens* produces several toxic secondary metabolites (e.g., hepatotoxic microcystins) that makes it a harmful species [4]. *P. rubescens* can regulate its vertical position in response to light [5] by producing or loosing gas vesicles and by accumulating or consuming dense carbohydrates. Further *P. rubescens* is able to cope with low light conditions and positions itself in the metalimnion [6] where it has access to increased levels of dissolved nutrients. The active buoyancy regulation minimizes sedimentation losses and allows for adjustment to moderate changes in stratification [7]. Buoyancy regulation thus may provide a competitive advantage of *P. rubescens* over other phytoplankton (e.g., green algae and diatoms). In spring when the lake re-stratifies, *P. rubescens* floats up to the metalimnion and subsequent population growth results in a dense metalimnic layer during summer [4], [8]. In autumn when stratification becomes weaker, events of deep vertical mixing can lead to surface blooms especially after metalimnic mass developments [8], [9]. During long-lasting periods of stable stratification, *P. rubescens* is able to out-compete other phytoplankton that suffers nutrient depletion in the upper mixed layer (epilimnion) and even dominates lake-wide phytoplankton biomass [2], [4].

Near-surface blooms of phytoplankton (in terms of Chl-*a*) and their spatial structure and temporal changes can be comprehensible measured by remote sensing techniques (airborne, satellites) [10], [11]. In contrast, these techniques are not able to detected deep chlorophyll maxima (DCM), as formed by e.g., *P. rubescens* during stratification of the water column. However, the spatial and temporal variations in a DCM may have important consequences for inter-specific competition in the phytoplankton community and for distribution patterns of organisms at higher trophic levels. In particular a metalimnic layer of toxic *P. rubescens* may interfere with the diel vertical migration of zooplankton, alter predator-prey interactions, and affect the distribution patterns of fish [12], [13], [14], [15].

A DCM (e.g., formed by *P. rubescens*) or phytoplankton in general is typically measured by water sample cell counts or *in situ* by fluorescence probes at single or multiple wavelengths, where the former is extremely time consuming and thus limited in its spatial and temporal resolution compared to the later. Zooplankton dynamics and distributions can be measured by acoustic devices, a common tool in oceanography and lake physics that are primarily used to measure horizontal and vertical current velocities and turbulence [16], [17], [18], [19], [20], after calibration of the acoustic backscatter strength (ABS) to the species that dominate the signal strength [21], [22], [23], [24]. As *P. rubescens* contains gas vesicles that imply a strong density difference and hence acoustic contrast to the ambient water, acoustic devices may be suitable to measure distributions and dynamics of *P. rubescens*.

This study presents and compares optical and acoustic *in-situ* techniques to measure spatial and temporal distribution patterns of plankton with special emphasis on *P. rubescens* in freshwater ecosystems. Specifically, we demonstrate that the combination of measurements with optical and acoustic *in-situ* sensors enables the qualitative and quantitative assessment of *P. rubescens* distributions and the distinction of *P. rubescens* from other phytoplankton and zooplankton.

## Materials and Methods

### Study site

The prealpine Lake Ammer is located in the southeast of Germany at an altitude of 553 m (47°59′N, 11°07′E). The lake is elongated in North-South direction (15 km length and 2–5 km width) with steep slopes along the western and eastern shores. Lake Ammer is a dimictic lake with a surface area of ∼47 km^2^, a maximum and mean depth of 81.1 m and 37.5 m, respectively. Lake Ammer is mainly fed by the River Ammer that enters the lake in the South (∼17 m^3^ s^−1^) and has a residence time of ∼2.7 years. Between the beginning of the 1970's and the middle of the 1990's Lake Ammer underwent a distinct phase of eutrophication (∼60 µg L^−1^ TP), followed by re-oligotrophication, and finally reaching again a mesotrophic state (∼10 µg L^−1^ TP) with a mean Secchi-depth of ∼3 m [4]. In contrast to the reduction of TP, the nitrogen concentrations remained high.

### Experimental design and instrumentation

**Figure 1:**
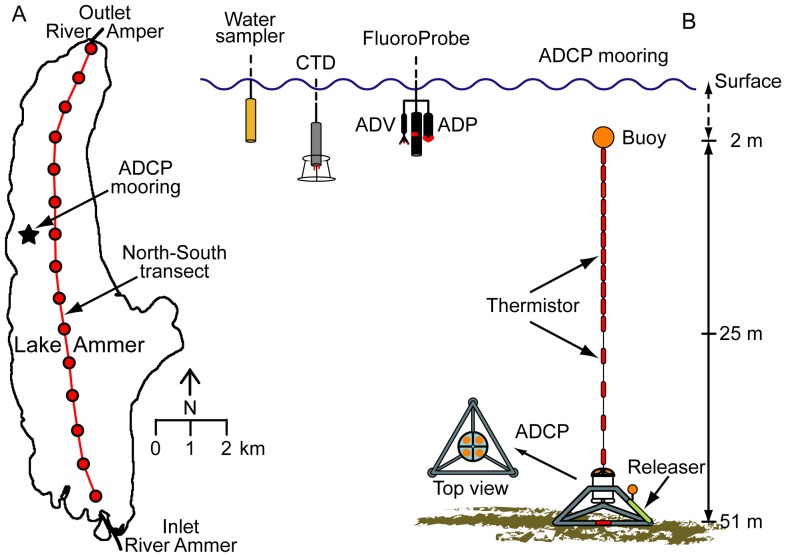
Study site and experimental setup A) Map of Lake Ammer including the position of the ADCP mooring with the thermistor chain (black star) and a North-South transect with 15 stations (gray dots) that had interspaces of ∼1 km. B) General configuration of the ADCP mooring with the thermistor chain and field devices used.

Measurements were conducted during two field campaigns in 2009 and 2011. In each of the years we surveyed a North-South transect with 15 sampling stations that had interspaces of ∼1 km (Fig. 1A; with permission of the Bavarian Lake Administration and the District Office Landsberg-Lech). At each of the stations we collected a vertical profile (from the water surface to at least 30 m water depth or to the bottom) with a multi-parameter probe measuring depth, temperature, conductivity (CTD-probe, RBR Ltd., Ottawa, Canada), turbidity (SEAPOINT SENSORS Inc., Exeter, NH), oxygen (fast optode 4330F, AANDERAA, Bergen, Norway), and chlorophyll-a (Seapoint Chlorophyll Fluorometer, SCF; SEAPOINT SENSORS Inc., Exeter, NH) and with a multi-spectral FluoroProbe (Moldaenke FluoroProbe, MFP; BBE MOLDAENKE, Schwentinental, Germany) that was conjointly lowered with two acoustic backscatter probes (ADP - 2 MHz in 2009 and 2011 and ADV - 6 MHz only in 2011, NORTEK, Rud, Norway; Fig. 1B). The CTD, MFP, ADP, and ADV sampled at frequencies of 6, 1, 1, and 8 Hz, respectively. Each profile (downcast) took 5–10 minutes corresponding to a vertical resolution of 0.02–0.1 m depending on the specific sampling frequency of the used device.

In 2009 a mooring equipped with a vertical thermistor chain and a bottom-resting 614 kHz acoustic Doppler current profiler (ADCP, Teledyne RD-Instruments, Poway, CA; Fig. 1B) was deployed between 21 August and 15 September at 51 m water depth in the north-west of Lake Ammer (Fig. 1, mooring marked with a black star). Vertically resolved water samples were taken three times at the position of the mooring in 2009 and at two stations of the North-South transect in 2011 to validate the *in-situ* optical and acoustical measurements of *P. rubescens*.

### Fluorescence (optical) measurements

Fluorescence measurements to determine the chlorophyll-*a* concentration of different algae groups and in particular of the cyanobacterium *P. rubescens* were conducted with two different devices: the standard fluorometer SCF and the multi-spectral FluoroProbe MFP. The SCF uses modulated blue LED lamps and a blue excitation filter to excite chlorophyll (excitation wavelength: 470 nm). The MFP is able to discriminate between four different spectral groups of algae (i.e., green group: Chlorophyta and Euglenophyta; blue-green group: Cyanobacteria; brown group - defined as ‘diatoms’: Heterokontophyta, Haptophyta, and Dinophyta; and mixed group - defined as ‘cryptophytes’: Cryptophyta) in mixed populations utilizing sequential light excitation by five LEDs emitting at 470, 525, 570, 590, and 610 nm [25]. Depending on the accessory pigments of the individual phytoplankton species a set of characteristic fingerprints (relative intensity of the fluorescence at the excitation wavelengths of 470, 525, 570, 590, and 610 nm) can be detected. The MFP software is then used to convert the recorded fluorescence values into the relative amount of each spectral algae group in terms of chlorophyll-*a* equivalents (µg Chl-*a* L^−1^) [25]. The factory calibrated fingerprint of the MFP for the ‘blue-green’ group (Cyanobacteria) accounts only for phycocyanin-containing cyanobacteria such as *Microcystis* sp., and not for ‘red’ cyanobacteria like *P. rubescens* that has a higher phycoerythrin content than phycocyanin-containing cyanobacteria [26], [27]. Therefore, the fluorescence signals of *P. rubescens* corresponds rather to the factory calibrated fingerprint of cryptophytes than to the factory calibrated fingerprint of the ‘blue-green’ group (Cyanobacteria), which was verified by lab experiments with *P. rubescens* isolates at chlorophyll-*a* concentrations between 4 and 26 µg L^−1^.

### Acoustic measurements

*In-situ* measurements of the acoustic backscatter strength (ABS) were conducted with three different acoustic backscatter devices: a 2 MHz high-resolution acoustic Doppler profiler (ADP, AQUADOPP HR - NORTEK, Rud, Norway), a 6 MHz acoustic Doppler velocity meter (ADV, VECTOR - NORTEK, Rud, Norway), and a 614 kHz acoustic Doppler current profiler (ADCP, Teledyne RD-Instruments, Poway, CA). These devices are commonly used to measure horizontal and vertical current velocities at a single depth (ADV) or as vertically resolved profile over the water column (ADP, ADCP). All three devices transmit sound at a fixed frequency (e.g., 614 kHz, 2 and 6 MHz) and listen to echoes returning from sound ‘scatterers’ (e.g., small particles or plankton) in the water. The ABS depends on the volume concentration, size, shape, and density of the scattering ‘particles’ [28]. The acoustic frequency of the specific device defines its sensitivity (target strength) to particles of different sizes [29], [30]. Target strength (TS) is a function of 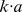, the acoustic wave number *k* times the particle radius *a* (
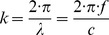
, where *f* denotes the frequency and *c* the phase speed in water). The maximum TS is reached at 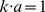, i.e., when the radius of the particle is equal to the acoustic wavelength divided by 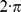. For particles with a radius smaller than 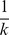, TS is proportional to the radius of the particle to the fourth power, whereas for particles with a radius larger than 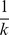, TS is inversely proportional the particle radius. Maximum TS of the ADCP, ADP, and ADV having acoustic frequencies of 0.614, 2, and 6 MHz are expected at particle diameters of ∼800, ∼240, and ∼80 µm, respectively. Thus, with increasing acoustic frequency, the maximum sensitivity of the acoustic devices is shifted to smaller particles.

The 614 kHz ADCP, which was deployed up-ward looking on a bottom-resting tripod at 51 m water depth Fig. 1B, was set up to measure horizontal and vertical current velocities and the received acoustic echo intensity (sound power per unit area received from the ‘scatterers’ in the water column) from 94 depth cells with a vertical resolution of 0.5 m, resulting in a profiling range from 2 m above the bottom to 3 m below the water surface. The ADCP averaged internally 20 individual profiles taken every 1.5 s thus providing a sampling internal of 30 s. The echo intensity received from the individual depth cells along the four beams was corrected for range dependent losses of acoustic power due to absorption in the water and beam spreading as described by Lorke et al. [31]. Thereafter, the resulting acoustic backscatter strength (ABS) from each of the depth cells of the four beams was averaged.

The 2 MHz ADP and the 6 MHz ADV (Fig. 1B), which were conjointly lowered (downward looking) with the MFP, were used to measure vertical profiles (only the downcast) of ABS at each of the sampling stations. The ADP has a short profiling range of 1 m ahead of the probe and received acoustic echo intensity from 20 depth cells with a vertical resolution of 0.05 m at 1 Hz sampling frequency. The echo intensity was converted into ABS as described for the ADCP (see above). The three depth cells 9–11 in the middle of the profiling range (0.5 m ahead of the transducer) were averaged to account for a potential bias of the ABS caused by tilting and swinging of the probe during lowering it and to reduce ringing effects in the near-field of the acoustic transducer. The pressure measured at the head of the acoustic transducer was corrected for the 0.5 m vertical spacing between the pressure sensor position and the mean depth cell range used for the data analysis. The ADV measured the ABS at a single, small sampling volume 0.15 m in front of the transducer at a sampling frequency of 8 Hz. The pressure measured at the head of the probe was corrected for the vertical spacing between the pressure sensor position and the sampling volume of the ADV. The time series of ABS measured with the ADP (ABS-ADP) and the ADV (ABS-ADV) were smoothed using a low-pass Butterworth filter (3 s cut-off period) to reduce the noise, which stems from tilting and swinging of the probes during the sensor lowering. The processing of the ADP and ADV data measured during down-casts provided vertical profiles of the ABS-ADP and ABS-ADV, respectively.

### Temperature measurements

The vertical thermistor chain deployed between 21 August and 15 September 2009 was equipped with 13 individual temperature loggers (TR-1060, RBR Ltd., Ottawa, Canada), which were located between 3 and 21 m with a vertical spacing of 2 m and at 30, 40, and 50 m water depth. The accuracy of the loggers is 0.002°C, and the sampling interval was set to 2 s. The temperature time series of each of the loggers was corrected for the occurring time lag (2–8 s) between the logger time at the end of the deployment and the host time by linear interpolation. The 5, 10, 15, and 20°C isotherms were calculated by linear interpolation of the vertical temperature profiles.

### *P. rubescens* cell densities

The *in-situ* optical and acoustical measurements were validated with *P. rubescens* cell densities that stem from five profiles taken at the position of the mooring (Fig. 1A) on 21 and 24 August 2009 (three profiles) and of the North-South-transect on 16 August 2011 (two profiles). Water samples were taken at 1, 4, 8, 10, 11, 12, 13, 14, 15, 20, and 25 m water depth using a 2-liter sampler (LIMNOS, Turku, Finland). Water samples (50 mL) were immediately fixed with Lugol's iodine solution and stored in darkness. Defined sample volumes were filtered on nitrocellulose membranes (pore size 8 mm, diameter 25 mm, Schleicher & Schuell, Germany). Filters were dried in darkness at room temperature and subsequently analyzed via fluorescence microscopy and image processing according to the protocol published in Ernst et al. [32]. The methodical error of this technique is ∼5% and depends on filament density.

### Zooplankton sampling

Zooplankton sampling was conducted parallel to the acoustical measurements at least twice in 2009 and 2011. Samples were collected between 0 and 50 m water depth within 5 to 10 m depth intervals using a closing net (Apstein-plankton net, opening diameter 17 cm, mesh size 100 µm, HYDRO-BIOS, Kiel, Germany). Zooplankton specimens were fixed in 4% sucrose-formaldehyde-solution for later taxon-specific counting under a dissecting microscope.

### Statistical analysis

Before plotting, the vertical profiles of all parameters measured with the CTD, MFP, ADP, and ADV were linear interpolated to the same distinct depth levels ranging from 0 to 30 m in steps of 0.05 m.

For the statistical comparison of the *in-situ* optical and acoustical data with *P. rubescens* cell densities measured in water samples, the profiles of the *P. rubescens* Chl-*a* equivalent concentration measured with the MFP (*P. rubescens*-MFP) and the profiles of the ABS-ADP and ABS-ADV were linear interpolated to the depths at which the water samples were collected. Least-square fit models (Eq. 1) were applied to evaluate the performance of the different techniques measuring *P. rubescens in situ* and to calibrate each of them to *P. rubescens* cell densities (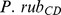). The following model was fitted to the data: 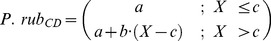
(1)where *X* stands for either the *P. rubescens*-MFP or the ABS-ADP and ABS-ADV. The model parameter *c* corresponds to the threshold value of *X*, above which the *in-situ* technique is sensitive to the cell densities of *P. rubescens*. Parameter *a* corresponds to the cell densities of *P. rubescens* below the threshold value and parameter *b* is the slope of the curve above the threshold value of *X*.

## Results

### Vertical distribution of *P. rubescens*

**Figure 2:**
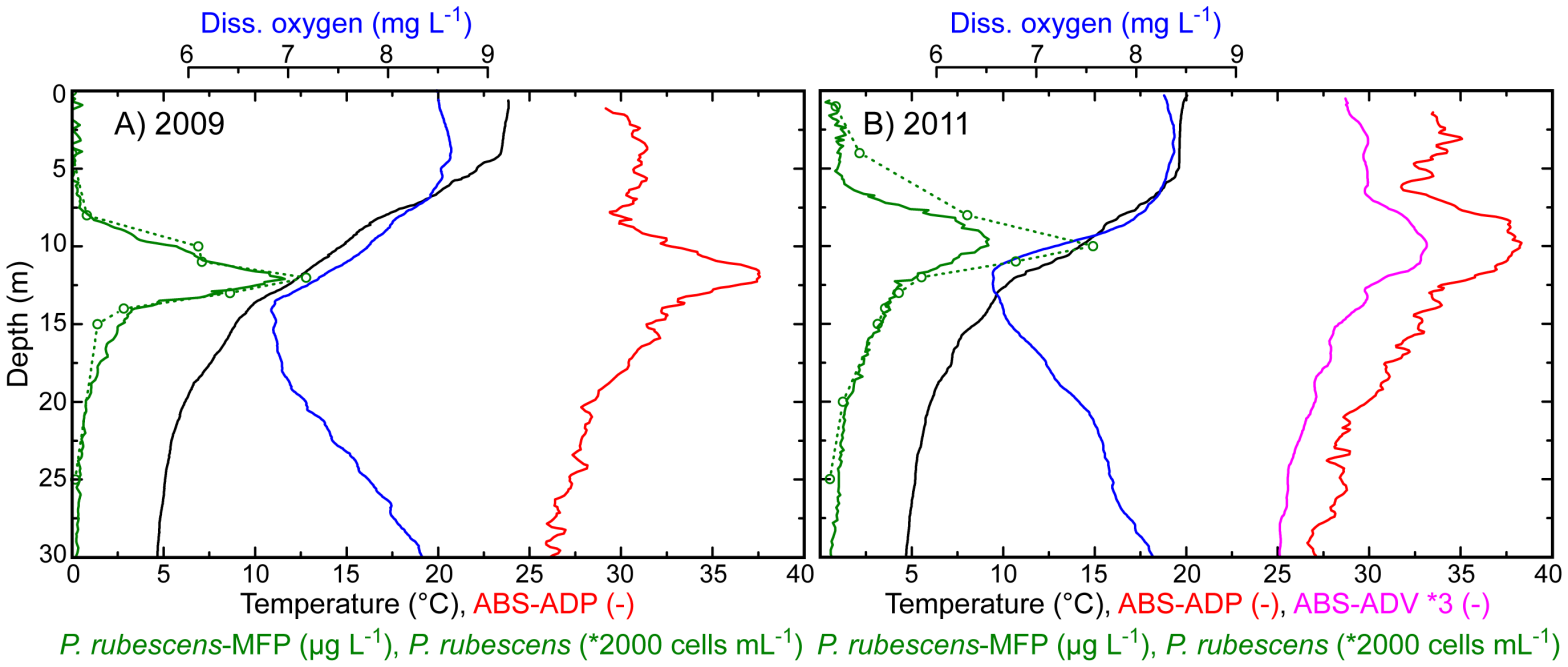
Vertical distribution of temperature (black), dissolved oxygen (blue), the acoustic backscatter strength ABS-ADP (red) and ABS-ADV (magenta), fluorescence in terms of *P. rubescens*-MFP (green solid line), and the cell density of the cyanobacterium *P. rubescens* (green open circles connected by a dotted line). The data were measured with a CTD-probe (temperature and diss. oxygen), two different acoustic backscatter probes (ADP - 2 MHz and ADV - 6 MHz), a Moldaenke FluoroProbe (*P. rubescens*-MFP), and microscope cell counts of water samples (density of *P. rubescens*), respectively, (A) on 21 August 2009 and (B) on 16 August 2011. The *in-situ* measurements had a vertical resolution of 0.02–0.10 m.

The vertical distribution of *P. rubescens* measured *in situ* by different optical (MFP) and acoustic (ADP and ADV) devices and the vertical profiles of *P. rubescens* cell densities measured in water samples are depicted in Figure 2 together with profiles of temperature and dissolved oxygen from the CTD. Figure 2 presents exemplary data sets from August 2009 and August 2011, respectively, measured at one station on the North-South-transect. *P. rubescens* was most abundant (±50% deviation from the peak concentration) at water depths between 10.1 and 13.4 m with a pronounced peak at 12.1 m in August 2009 and between 7.5 and 12.5 m with a pronounced peak at 9.8 m in August 2011.

In both years *P. rubescens* formed a layer of high cell densities in the metalimnion, where maximum densities occurred in the region of the highest density (temperature) gradient. Maximum cell densities reached values of ∼26,000 and ∼29,000 cells mL^−1^ in 2009 and 2011, respectively. Dissolved oxygen was gradually depleted in the metalimnic layer down to 6.5–7 mg L^−1^ at ∼2–3 m below the *P. rubescens* peak (Fig. 2).

The *P. rubescens*-MFP had the same structure in the vertical distribution as the *P. rubescens* cell densities (Fig. 2). Especially for the data set from 2009, in which the vertical distribution of the *P. rubescens* peak is better resolved by water samples than in the data set from 2011, *in-situ* measurements agree well with details of the vertical distribution of *P. rubescens* cell densities. The *in-situ* data suggest that the metalimnic *P. rubescens* layer extended over a larger depth range in 2011 than in 2009 (3.3 and 5.0 m, respectively, ±50% deviation from the peak concentration).

The vertical profiles of ABS-ADP and ABS-ADV both show a distinct metalimnic peak at the same depth range as the vertical profiles of *P. rubescens*-MFP and the *P. rubescens* cell densities measured in water samples (Fig. 2). The ABS-ADP and ABS-ADV values in the epilimnion (upper mixed layer of the water column) are typically larger than in the hypolimnion indicating that the volume concentration of other acoustic ‘scatterers’, e.g., suspended sediments, was lower in the hypolimnion than in the epilimnion. Nevertheless, ABS-ADP and ABS-ADV profiles not only share the feature of a distinct metalimnic maximum with the profiles of *P. rubescens*-MFP and *P. rubescens* cell densities but also have a similar vertical structure. Corresponding to the measurements with the MFP and the water sample cell counts, indicating a larger vertical extent of the metalimnic *P. rubescens* peak in 2011 than in 2009, ABS-ADV and ABS-ADP profiles both show that the metalimnic layer of elevated ABS was larger in 2011 than in 2009.

### *In-situ* calibration of *P. rubescens* Chl-*a* equivalents and acoustic backscatter strength to *P. rubescens* cell densities

**Figure 3:**
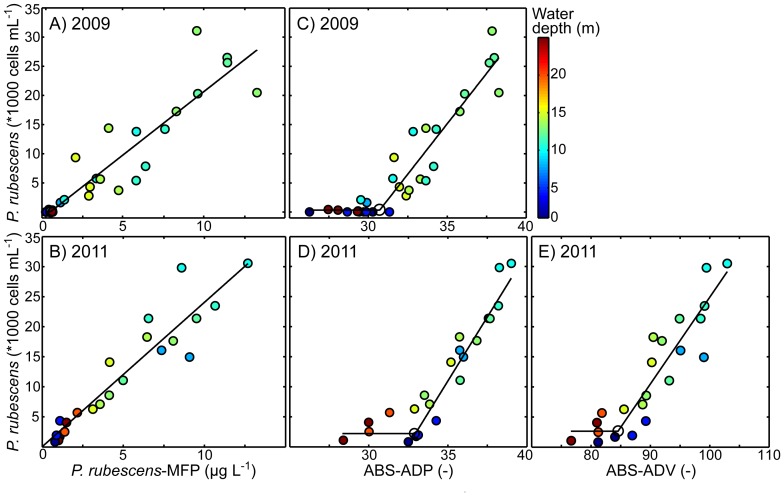
Scatter plots of the *P. rubescens* cell densities and (A–B) the *P. rubescens* Chl-*a* equivalent concentration measured with the Moldaenke FluoroProbe (*P. rubescens*-MFP), (C–D) the acoustic backscatter strength ABS measured with the ADP (ABS-ADP), and (E) the acoustic backscatter strength ABS measured with the ADV (ABS-ADV), respectively. The data stems from profiles collected along the North-South transect. For 2009 three profiles were available measured on the 21 and 24 August and for 2011 two profiles were available from the 16 August 2011. Water samples for the analysis of *P. rubescens* cell densities were taken at 1, 4, 8, 10, 11, 12, 13, 14, 15, 20, and 25 m water depth. The profiles of the *P. rubescens*-MFP, the ABS-ADP, and the ABS-ADV were linear interpolated to the water sample depths, respectively. The black line represents the results of the least-squares fit model (Eq. 1).

The similarities in the vertical distribution of *P. rubescens* cell densities measured in water samples and *P. rubescens*-MFP as well as the ABS-ADP and ABS-ADV (Fig. 2) suggest a strong correlation between the *P. rubescens* concentrations obtained with *in-situ* optical and acoustic techniques and cell counting in water samples. This conclusion is confirmed in Figure 3 depicting the correlation between data from water samples and *in-situ* measurements for all data sets that include cell counts from water samples. The *P. rubescens* cell densities from 2009 and 2011 correlate well with *P. rubescens*-MFP and ABS, and the least-squares fit model (Eq. 1), applied to calibrate the data from the *in-situ* devices, provides similar calibration parameters for the two years (Fig. 3A–D and Table 1).

**Table 1:** Least-squares fit parameters and significance (Eq. 1) of the *in-situ* calibration of *P. rubescens* Chl-*a* eq. concentration (µg L^−1^) and acoustic backscatter strength ABS (-) to *P. rubescens* cell densities (cells mL^−1^) measured with MFP, ADP, and ADV, respectively in 2009 and 2011.

*In-situ* technique	Fit parameter Eq. 1		2009	2011
	*a*	(cells mL^−1^)	98	1
	*b*	(cells mL^−1^ µg^−1^ L)	2139	2401
Chl.-*a* - MFP	*c*	(µg L^−1^)	0.3	0.0
	*R^2^*	(-)	0.85	0.88
	*p*	(-)	<0.0001	<0.0001
	*a*	(cells mL^−1^)	389	2169
	*b*	(cells mL^−1^)	3461	4266
ABS-ADP	*c*	(-)	30.7	32.9
	*R^2^*	(-)	0.82	0.92
	*p*	(-)	<0.0001	<0.0001
	*a*	(cells mL^−1^)	-	2636
	*b*	(cells mL^−1^)	-	1441
ABS-ADV	*c*	(-)	-	84.4
	*R^2^*	(-)	-	0.81
	*p*	(-)	-	<0.0001

*P. rubescens*-MFP are significantly correlated with the *P. rubescens* cell densities (Fig. 3A,B and Table 1). The least-squares fit parameters indicate that *P. rubescens*-MFP and *P. rubescens* cell densities (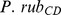; Eq. 1) are linear correlated having an intercept close to zero and thus could also be described by a linear regression model. The linear slope differed only slightly between 2009 and 2011 (Table 1, parameter B). The low abundances of *P. rubescens* were measured at large and shallow water depths whereas the high abundances were from intermediate water depths, where the *P. rubescens* layer was located (Fig. 3A,B, color coding).

For ABS-ADP and ABS-ADV below a certain threshold value cell densities of *P. rubescens* remain rather constant, but above this threshold level *P. rubescens* cell densities increase in close correlation with increasing ABS (Fig. 3C–E). This indicates that a threshold value of ABS is required to discriminate between *P. rubescens* and other ‘scatterers’ but that above this threshold value ABS is an excellent measure of concentrations of *P. rubescens* (Table 1). The ADP is sensitive to *P. rubescens* above a threshold ABS of ∼31 corresponding to *P. rubescens* cell densities of ∼500 cells mL^−1^. The threshold value slightly differed between 2009 and 2011. The ADV measured higher absolute ABS than the ADP because of the design of its transducer. The ADV is sensitive to *P. rubescens* above a threshold ABS of ∼84 corresponding to *P. rubescens* cell densities of ∼2,500 cells mL^−1^. Thus, compared to the ADV the ADP has a slightly higher sensitivity to low *P. rubescens* cell densities.

### Spatial distribution of *P. rubescens* in relation to Chl-*a*, diatoms, and abiotic parameters

**Figure 4:**
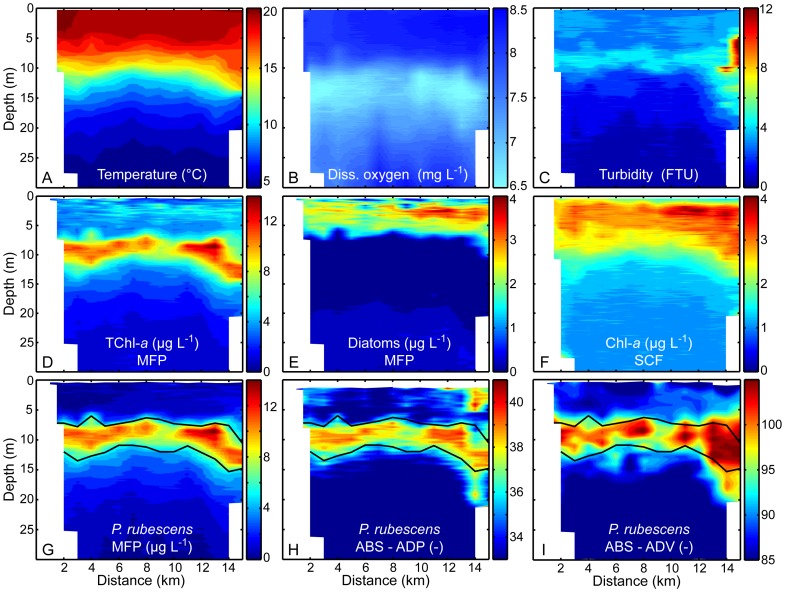
Spatial distribution of physical parameters and different algae groups along a North-South directed transect at Lake Ammer on 16 August 2011. (A) Temperature (°C). (B) Dissolved oxygen (mg L^−1^). (C) Turbidity (FTU). (D, E, and G) Concentration (µg L^−1^) of total chlorophyll-*a* (TChl-*a*), diatoms, and *P. rubescens* (Moldaenke FluoroProbe, MFP). (F) Concentrations of chlorophyll-*a* (µg L^−1^) measured with the Seapoint Chlorophyll Fluorometer (SCF). (H–I) Acoustic backscatter strength (ABS) measured with the ADP (ABS-ADP) and the ADV (ABS-ADP). The black solid lines in panels G–I indicate the 50% deviation from the local peak concentration of the *P. rubescens* Chl-*a* equivalent concentration (panel G) at each station measured with the MFP (*P. rubescens*-MFP). The transect had a spatial resolution of 1 km along the center line of the lake.

The spatial distributions of *P. rubescens*, diatoms, Chl-*a*, and abiotic parameters (e.g., temperature, diss. oxygen, and turbidity) were measured with the different *in-situ* optical and acoustic devices and the CTD probe along a North-South directed transect at Lake Ammer (Fig. 4). The transect depicted in Figure 4 is from the 16 August 2011, when both acoustic devices (ADP and ADV) were also deployed. Lake Ammer was stable stratified with a warm, homotherm epilimnion (∼19°C down to ∼7 m), a metalimnion that was located between 7 and 16 m (temperature gradient >1°C m^−1^), and a cold hypolimnion below 16 m depth (4–6°C) (Figs. 2 and 4A). *P. rubescens* formed a dense metalimnic layer with Chl-*a* eq. concentrations (MFP) up to 14 µg L^−1^ below which dissolved oxygen was depleted (at 10–20 m) (Fig. 4B,G). The thickness of the *P. rubescens*-MFP layer was on average 5 m (±50% deviation from the local peak concentration). All along the transect, the depth range of the layer of elevated values for *P. rubescens*-MFP agrees excellently well with the depth range of the layer of elevated ABS-ADP and ABS-ADV (Fig. 4G–I). Also the mean thickness of the ABS-ADV and the ABS-ADP layer agrees well with the mean thickness of the *P. rubescens*-MFP layer. This comparison illustrates that the vertical distributions of *P. rubescens* inferred from *in-situ* data measured with ADP and ADV not only agree locally with those from MFP but also at numerous locations in the lake. This suggests that the acoustic techniques are similar well suitable as the optical technique to assess spatial distributions of *P. rubescens*.

The MFP data indicate that diatoms were present in the epilimnion of Lake Ammer (Fig. 4E) above the distinct metalimnic layer of *P. rubescens*. The Chl-*a* eq. concentration of diatoms ranged between 1 and 4 µg L^−1^ with highest concentrations in the South, where River Ammer enters the lake (Figs. 1A and 4E,G). The Chl-*a* eq. concentration of diatoms were far below the concentrations measured for *P. rubescens*. Concentrations of the other algae groups obtained from the MFP were negligible. *P. rubescens* was the most abundant phytoplankton species and dominated the distribution of total Chl-*a* in Lake Ammer at that time of the year (linear regression between total Chl-*a* and *P. rubescens* Chl-*a* eq. concentration, *R^2^* = 0.82, *p*<0.0001).

The spatial distribution of the Chl-*a* concentration measured with the commonly used Seapoint Chlorphyll Fluorometer (Chl-SCF) along the North-South directed transect closely corresponds to the distribution of the diatoms (MFP) that were restricted to the epilimnic layer (Fig. 4E,F). The Chl-SCF ranged between 2 and 4 µg L^−1^ with highest concentrations in the South as observed for the diatoms (MFP). In the metalimnion, the Chl-SCF was ∼1 µg L^−1^ suggesting that the SCF is not sensitive to the abundance of *P. rubescens*.

### Spatial and temporal distribution and vertical motion of *P. rubescens* and zooplankton

**Figure 5:**
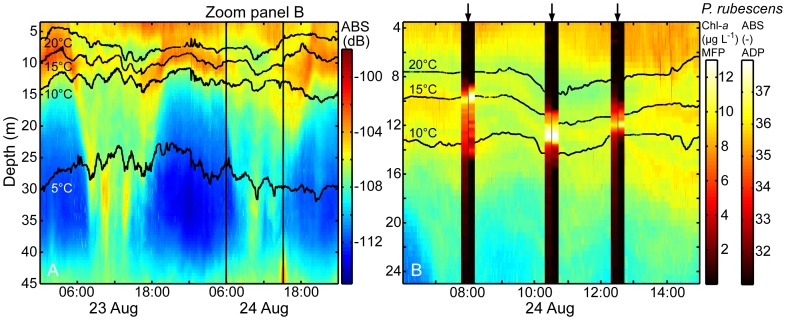
Temporal changes in the vertical distribution of *P. rubescens* and zooplankton between the 23 and 24 of August 2009. (A) Contour plot of the acoustic backscatter strength (ABS-ADCP) and the 5, 10, 15, and 20°C isotherms (black solid lines) between 3.5 and 45 m water depth. (B) Temporal (24 August 2009 between 06:00 and 15:00 h) and spatial (depth range: 3.5 and 25 m) zoom in of panel A with additional profiles (black arrows) of the vertical distribution of *P. rubescens* in terms of Chl-*a* equivalent concentration (left hand side, (*P. rubescens*-MFP) and acoustic backscatter strength (right hand side, ABS-ADP). The ABS-ADCP was measured with a bottom-mounted 614 kHz ADCP. Isotherms were calculated from the data of the thermistor chain.

Repeated profiling with jointly lowered MFP and ADP (optical and acoustical measurements of *P. rubescens*) combined with temporally highly-resolved vertical profiles of ABS measured at 614 kHz with a bottom-mounted ADCP (ABS-ADCP) reveals details on the vertical distribution and motion of the *P. rubescens* layer and zooplankton (Fig. 5). The vertical profiles from the *P. rubescens*-MFP (Fig. 5B) and the ABS-ADP (2 MHz) have essentially the same vertical structure and indicate that *P. rubescens* was abundant between 9 and 15 m water depth (Fig. 5B), which confirms the results of the previous sections (Figs. 2 and 4). The ABS measured with the ADCP at 614 kHz also show a band of increased ABS-values at this depth range and appears to be sensitive to *P. rubescens* at day time (Fig. 5). On 24 August between 08:00 and 13:00 h, the band of elevated ABS between 9 and 15 m was subdivided into 2–3 separate bands (sub-bands) where each of them had a vertical extent of ∼1 m (Fig. 5B). The peak values of the *P. rubescens*-MFP and the ABS-ADP coincide with the central sub-band. The upper and the lower sub-bands frame the vertical extent of the metalimnic *P. rubescens* layer. The time series of the profiles measured with the ADCP is additionally characterized by daily cycles in the ABS in the deep water indicating diel vertical migration of zooplankton (Fig. 5A). Zooplankton sampling confirmed the presence of copepods in the deep water and *Daphnia* next to the water surface at day time. At night, zooplankton and *P. rubescens* co-occured in the near-surface layer (down to 12 m) and thus cannot be discriminated by the ABS-ADCP (Fig. 5A). The vertical distributions of both, *P. rubescens* and zooplankton, followed the vertical displacement (∼3 m) of the isotherms (Fig. 5).

## Discussion

### Advantages and limitations of *in-situ* fluorescence measurements of *P. rubescens* and other phytoplankton

The MFP provides a fast and reliable technique to measure fluorescence at different wavelengths *in situ*, which allows the discrimination of four different algae groups or of specific species, e.g., the buoyant and potentially harmful cyanobacterium *P. rubescens*. The high correlation between the *in situ* measured *P. rubescens*-MFP and the microscopic cell counts (cell density) of *P. rubescens* in Lake Ammer from two different years reveals the qualitative and quantitative reliability of this technique (Figs. 2 and 3; Table 1) and further supports earlier studies using the MFP for this purpose [27], [33]. Thus, multi-spectral *in-situ* fluorescence measurements are a reliable alternative to the time consuming microscopic counting, if the focus of interest is on the dominant algae species or groups in the lake ecosystem. However, the technique requires regular and independent determination of the algal community from water samples to identify changes in the composition of the dominant species that may affect the assignment of the pre-defined optical fingerprints to algal groups.

Particular difficulties arise if two different dominant phytoplankton species or groups have similar fingerprints. For example, *P. rubescens* and cryptophytes both contain phycoerythrin as main accessory pigment and therefore have very similar and almost linear dependent norm spectra. A distinction between *P. rubescens* and cryptophytes based on differences in the fluorescence signals at the five wavelengths measured with the MFP is therefore very unreliable and associated with very large errors in the abundances estimated by linear decomposition of the five fluorescence signals [25]. In lakes where *P. rubescens* and cryptophytes co-occur (e.g., Lake Pusiano) [26], the concentration estimates (Chl.-*a* eq.) are associated with large uncertainties even if individual fingerprints are provided. The distinction is then based on fluorescence channels with small signals that may also be affected by the presence of other species with accessory pigments fluorescing at these wavelengths (e.g., phycoerythrin-rich cyanobacteria) [26].

The Chl-SCF corresponded to the Chl-*a* eq. concentration of diatoms (MFP) in the upper mixed layer of the water column (Fig. 4E,F). Both measures showed the same horizontal distribution characterized by higher concentrations in the South of Lake Ammer, where River Ammer enters the lake, increases the nutrient content [34], and thus favors differential and preferential growth of diatoms. The Chl-*a* eq. concentration of *P. rubescens* did not contribute to the Chl-SCF. Apparently, the SCF using an excitation wavelength of 470 nm is not sensitive to algae with high phycoerythrin content and thus does not detect the metalimnic layer formed by the toxic cyanobacterium *P. rubescens* (Fig. 4F,G), which usually dominates the phytoplankton community in Lake Ammer during summer and autumn [4].

### Advantages and limitations of *in-situ* acoustical measurements of plankton

*In-situ* measurements of the ABS conducted with three different acoustic devices covering multiple acoustic frequencies (614 kHz ADCP, 2 MHz ADP, and 6 MHz ADV) were tested for their ability to measure the spatial and temporal distribution of the cyanobacterium *P. rubescens* under the presence of other zooplankton in Lake Ammer.

All three different acoustic devices that measured the ABS at three different acoustic frequencies were sensitive to *P. rubescens*. The ABS-ADP and ABS-ADV was even dominated by *P. rubescens* as is indicated by the high ABS-values in the metalimnion, which were in accordance with the vertical distribution of the *P. rubescens*-MFP and *P. rubescens* cell densities (Figs. 2 and 4G–I). The ABS-ADP and ABS-ADV are very well correlated to the *P. rubescens* cell densities above a device-specific ABS background level that corresponds to comparatively low *P. rubescens* cell densities (Fig. 3; Table 1). Hence, the ADP and the ADV that have their maximum target strength (TS) at ∼240 and ∼80 µm, respectively, are highly sensitive to *P. rubescens* with mean filament sizes of 100–500 µm, but are not sensitive to particles that are in the range of large meso-zooplankton (>500 µm). This conclusion is supported by the measurements during daytime (Fig. 5B). The vertical distribution of ABS-ADP correlates very well with the vertical distribution of *P. rubescens*-MFP, but the high ABS measured with the ADCP at shallow depths indicating the presence of zooplankton is not present in the ABS-ADP profiles.

The ADCP allows stationary measurements of the ABS with a high vertical resolution on times scales from seconds to seasons. In the last decade, the ABS-ADCP was frequently applied to observe the dynamics of large zooplankton (≥1 mm; e.g., *Daphnia* and *Chaoborus* larvae) in freshwater after calibration [22], [31], [35]. The maximum TS of the 614 kHz ADCP is at ∼800 µm, which favors detection of the large zooplankton if the density contrast between large and small zooplankton is marginal, which appears to be often the case [22]. Thus, if the TS of co-occurring zooplankton are similar, species differentiation is not possible using a single frequency. Although *P. rubescens* is smaller than the abundant meso-zooplankton its TS is similar to that of the zooplankton because the gas vesicles of *P. rubescens* lead to a strong acoustic contrast compared to the ambient water. During daytime, *P. rubescens* is located in the metalimnion of Lake Ammer whereas *Daphnia* stays in the upper mixed layer and copepods have emigrated to the hypolimnion (diel vertical migration of copepods in Lake Ammer, taxon-specific data not shown) (Fig. 5). Hence, the ABS from the ADCP at different depths can be associated with the different zooplankton groups and *P. rubescens*. At night, all zooplankton and *P. rubescens* co-occur in the epi- and metalimnion (Fig. 5A). Then it is impossible to distinguish between the different plankton groups and to estimate their concentrations from the ABS of the ADCP alone. Supplemental measurements with the ADP or ADV providing estimates of the concentrations of *P. rubescens* could be used to disentangle *P. rubescens* from zooplankton in the ABS-ADCP at night.

Beside zooplankton, the concentration of other ‘scatterers’ like sediment particles can additional contribute to the ABS and thus influence the qualitative and quantitative estimates on *P. rubescens* from ABS, as is the case for estimates of zooplankton too [36], [37]. Particles that are suspended in the open water of lakes are typically characterized by small sizes of a few micrometers and do not significantly affect the ABS at low turbidity (<5 FTU; Fig. 4C,H,I). But next to river estuaries, where high turbidity can occur and particle sizes can be within the range of the maximum TS of the acoustic frequencies used in this study, suspended particles reduce the significance and reliability of the ABS (ADP and ADV) as a measure of *P. rubescens* (Fig. 4C,H,I).

It is likely that all three different acoustic devices (ADCP and especially ADP and ADV) are not only sensitive to *P. rubescens*, but also to other filamentous, gas vesicle-producing cyanobacteria (e.g., *Arthrospira*, *Pseudoanabaena*, *Limnoraphis*, and *Calothrix*). This makes acoustic devices broadly applicable for *in-situ* measurements of this group of cyanobacteria. In the case of co-occurrence, differentiation between the different species may be rather difficult by acoustic measurements alone, but may be possible if the acoustic measurements are combined with optical *in-situ* techniques, e.g., the MFP.

### Capability and use of combined *in-situ* optical and acoustical measurements

Combined *in-situ* optical and acoustical measurements allow distinguishing between *P. rubescens* (and maybe other filamentous, gas vesicle-producing cyanobacteria), other phytoplankton, and zooplankton and assessment of plankton abundances at high temporal (minutes) and spatial (centimeters) resolution over large temporal (seasons) and spatial (basin-wide) scales. Especially in lakes where *P. rubescens* and cryptophytes co-occur (e.g., Lake Pusiano) [26], the discrimination between both may be uncertain if it is solely based on MFP data. A more reliable measure of cryptophytes and *P. rubescens* may be obtained by combining the MFP with acoustic devices (ADP and ADV), whereby the MFP provides a composite measure of cryptophytes and *P. rubescens* but the ADP and ADV are sensitive only to *P. rubescens* because of its gas vesicles.

The combination of optical and acoustic instruments can be further supplemented with CTD probes (multi-parameter probes) measuring abiotic parameters such as temperature, dissolved oxygen, turbidity, and pH. Such a combined instrument package can be used for stationary as well as vessel-mounted observations of plankton dynamics and abiotic conditions, where the former focuses on the vertical and the latter on the horizontal dimension.

In lake ecosystems horizontal and vertical gradients in species abundances are caused by heterogeneous resource availability, physical forcing, and organismic interactions [38], [39], [40], [41]. The combination of the optical and acoustical *in-situ* measurement techniques with water sample analysis (e.g., phytoplankton cell counts) and CTD probes provides an excellent basis for the investigation of the implications of currents and mixing on the horizontal and vertical distribution patterns of plankton communities, the role of bays and river inflows for generating horizontal heterogeneities (patchiness) in plankton, and the interaction between diel vertical migration of zooplankton and the phytoplankton community.
